# Dramatic switchable polarities in conduction type and self-driven photocurrent of BiI_3_ via pressure engineering

**DOI:** 10.1093/nsr/nwae419

**Published:** 2024-12-03

**Authors:** Lei Yue, Fuyu Tian, Ran Liu, Zonglun Li, Ruixin Li, Chenyi Li, Yanchun Li, Dongliang Yang, Xiaodong Li, Quanjun Li, Lijun Zhang, Bingbing Liu

**Affiliations:** State Key Laboratory of Superhard Materials, Jilin University, Changchun 130012, China; Key Laboratory of Automobile Materials of MOE and School of Materials Science and Engineering, Jilin University, Changchun 130012, China; State Key Laboratory of Superhard Materials, Jilin University, Changchun 130012, China; State Key Laboratory of Superhard Materials, Jilin University, Changchun 130012, China; State Key Laboratory of Superhard Materials, Jilin University, Changchun 130012, China; State Key Laboratory of Superhard Materials, Jilin University, Changchun 130012, China; Beijing Synchrotron Radiation Facility, Institute of High Energy Physics, Chinese Academy of Sciences, Beijing 100049, China; Beijing Synchrotron Radiation Facility, Institute of High Energy Physics, Chinese Academy of Sciences, Beijing 100049, China; Beijing Synchrotron Radiation Facility, Institute of High Energy Physics, Chinese Academy of Sciences, Beijing 100049, China; State Key Laboratory of Superhard Materials, Jilin University, Changchun 130012, China; Key Laboratory of Automobile Materials of MOE and School of Materials Science and Engineering, Jilin University, Changchun 130012, China; State Key Laboratory of Superhard Materials, Jilin University, Changchun 130012, China

**Keywords:** high pressure, conduction-type switching, self-driven photocurrent, metal halide

## Abstract

The intentional manipulation of carrier characteristics serves as a fundamental principle underlying various energy-related and optoelectronic semiconductor technologies. However, achieving switchable and reversible control of the polarity within a single material to design optimized devices remains a significant challenge. Herein, we successfully achieved dramatic reversible p–n switching during the semiconductor‒semiconductor phase transition in BiI_3_ via pressure, accompanied by a substantial improvement in their photoelectric properties. Carrier polarity flipping was monitored by measuring the photocurrent dominated by the photothermoelectric (PTE) effect in a zero-bias two-terminal device. Accompanying the p–n transition, a switch between positive and negative photocurrents was observed in BiI_3_, providing a feasible method to determine the conduction type of materials via photoelectric measurements. Furthermore, the combined effects of the photoconductivity and PTE mechanism improved the photoresponse and extended the detection bandwidth to encompass the optical communication waveband (1650 nm) under an external bias. The remarkable photoelectric properties were attributed to the enhanced energy band dispersion and increased charge density of BiI_3_ under pressure. These findings highlight the effective and flexible modulation of carrier properties through pressure engineering and provide a foundation for designing and implementing multifunctional logic circuits and optoelectronic devices.

## Teaser

This study reports a dramatic reversible p–n switching in BiI3 under pressure and proposes a feasible method to determine the conduction type of materials via photoelectric measurements.

## INTRODUCTION

Our ability to master semiconductor technology depends on controlling the type and density of electrical carriers to design optimized devices. Achieving a comprehensive understanding and precise control of carrier transport behavior profoundly influences the stability and power efficiency of forthcoming large-scale integrated circuits [1]. For digital-analog applications of 2D semiconductors, optimizing the functional integration of electronics and logic circuits remains a critical challenge. Achieving conduction-type transitions within a single material represents a crucial step in this endeavor [2,3]. Currently, the majority of 2D semiconductors, including MoS_2_ [4], WSe_2_ [5], and others, exhibit predominantly unipolar conduction, either electron-dominated n-type or hole-dominated p-type behavior. Tremendous efforts have been dedicated to achieving conduction behavior transitions, such as through doping engineering [6], material alloying [7], and heterojunction construction [8]. However, multipolar transitions within a single material have rarely been reported.

High pressure is an alternative external stimulus to temperature that can efficiently modify the crystal structure and electronic configuration, making it a powerful technique for altering physical and chemical properties [9–12]. The application of pressure has facilitated the discovery of various novel physical properties, including spin crossover [13], piezochromism [14], and metallization [15], which are similar to those observed under temperature variation. Therefore, pressure-driven conduction type switching should not be viewed as an exception. Actually, pressure-induced p–n switching has been observed in several narrowband semiconductor systems [16–24]. One class of these transitions primarily arises from electronic transitions, often appearing as Fermi surface topological changes known as Lifshitz transitions, as observed in materials such as Bi_2_Te_3_ [22]. The other class is associated with pressure-induced structural phase transitions, which are found in materials such as CuFeS_2_ [16], CrSb_2_ [17], and Cr_2_Se_3_ [18]. However, the p–n switching in these materials is typically accompanied by a semiconductor‒metal transition, which significantly limits their application in fields such as photodetection [17–22]. Therefore, achieving rapid carrier-type switching through a semiconductor‒semiconductor phase transition in functional semiconductors with suitable bandgaps is critically important. This advancement could enable the development of pressure-driven p–n switches or the fabrication of single-component n–p or n–p–n junctions through the application of external stress.

Bismuth triiodide (BiI_3_) has emerged as a key layered material within the metal halide family. Owing to its distinctive electronic structure and properties, it is an appealing candidate for various applications, including gamma/X-ray detectors [25], high-efficiency photovoltaic absorbers [26], and energy storage devices [27]. Metal halides typically feature soft lattices, so their structures and properties can be significantly altered by applying pressure. To date, pressure-induced changes in various physical properties, such as enhancement of the carrier concentration [28], light emission [29], and reduction of the bandgap [30], have been widely achieved in metal halide materials. Research has shown that a structural phase transition occurs in BiI_3_ at ∼5 GPa, primarily driven by changes in the coordination number. Additionally, the bandgap of BiI_3_ consistently decreases with increasing pressure, transitioning from a semiconductor to a metallic state above 35 GPa [31]. These phenomena are advantageous for manipulating the photoelectric properties of BiI_3_ through pressure, enabling a comprehensive exploration of the structure-property relationships to optimize its photoelectric characteristics.

In this study, we report dramatic reversible p–n switching during the semiconductor‒semiconductor phase transition in BiI_3_ under pressure, accompanied by a substantial improvement in the photoelectric properties. Comprehensive high-pressure characterization was employed to investigate variations in structural and physical properties. Furthermore, first-principles calculations demonstrated that the improved photoelectric properties of BiI_3_ under pressure originated from the increased energy band dispersion and enhanced charge density.

## RESULTS AND DISCUSSION

**Figure 1.**
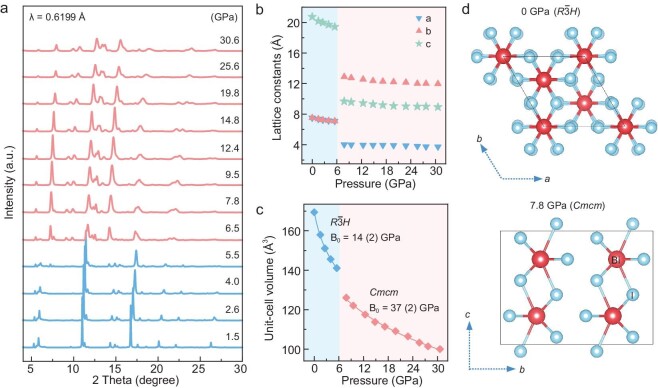
Structural evolution of BiI_3_ under high pressure. (a) *In situ* ADXRD patterns of BiI_3_ at various pressures. (b) Cell parameters and (c) unit-cell volume of BiI_3_ as a function of pressure. The curves are fitted using the Birch–Murnaghan equation of state. (d) Crystal structures of BiI_3_ at ambient pressure and 7.8 GPa.

High-quality BiI_3_ single crystals were sourced from Alfa Aesar. The uniform distribution of elements in the BiI_3_ crystals was confirmed through scanning electron microscopy (SEM) coupled with energy-dispersive X-ray spectroscopy (EDS), as illustrated in Fig. S1. Under ambient conditions, BiI_3_ is a layered semiconductor composed of honeycomb layers formed by edge-sharing BiI_6_ octahedra. To determine the structural variations in BiI_3_ under high pressures, comprehensive *in situ* angle-dispersive synchrotron X-ray diffraction (ADXRD) experiments were performed up to 30.6 GPa (Fig. 1a). Below 5.5 GPa, all the diffraction peaks shift to higher angles with increasing pressure owing to lattice contraction and are effectively indexed to a trigonal crystal structure with the *R*$\bar{3}$*H* space group. With further compression, several new diffraction peaks emerge, indicating a pressure-induced structural phase transition. Figure S2 displays the Rietveld analysis results for typical ADXRD data. The crystallographic analysis at 7.8 GPa confirms an orthorhombic structure (*Cmcm*), consistent with previously reported findings [31]. The dynamic changes in the lattice parameters and volumes with varying pressure are shown in Fig. 1b, c, and Table S1. In the *R*$\bar{3}$*H* phase, the lattice parameter along the *c*-axis decreases more significantly than those along the *a*- and *b*-axes, indicating larger compressibility along the interlayer direction. The *Cmcm* phase of BiI_3_ also exhibits anisotropic compression behavior, with the compression rates along the *b*- and *c*-axes slightly higher than that along the *a*-axis. The pressure–volume curves were fitted by the Birch–Murnaghan equation of state [32]. The parameters derived from this fitting reveal that the *Cmcm* phase with *B*_0_ = 37 (2) GPa is considerably more rigid than the *R*$\bar{3}$*H* phase with *B*_0_ = 14 (2) GPa (Fig. 1c). The structural transformation is represented in Fig. 1d and Fig. S3, which effectively show the crystal structure of BiI_3_ at 0 and 7.8 GPa. During the structural transition of BiI_3_, the coordination number of bismuth increases from 6 to 8, accompanied by a volume reduction of ∼10.6%. This results in higher packing density, which is consistent with the pressure-distance paradox and the pressure-coordination rule [31]. Notably, concomitant with the structural phase transition, various physical properties of the material potentially significantly change. Designing and precisely controlling the physical properties associated with phase transitions is pivotal in the development of modern functional materials.

**Figure 2.**
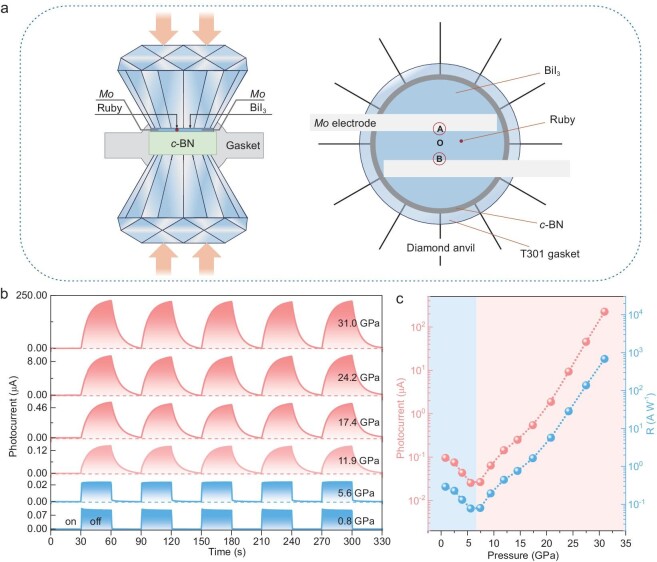
Photocurrent measurements of BiI_3_ under high pressure. (a) Schematic of the device used for *in situ* high-pressure photoelectric measurements. (b) Photocurrent curves of BiI_3_ under xenon light illumination at typical pressures with a 5 V bias. The light spot size greatly exceeds the area of the active device, resulting in global irradiation. (c) Pressure-dependent photocurrent and responsivity (R) of BiI_3_, derived from the data in (b).

To comprehensively investigate the photoelectric characteristics of BiI_3_ under compression, we developed an *in situ* high-pressure photoelectric measurement setup employing a two-point probe method within a diamond anvil cell (DAC). A schematic diagram of this setup is shown in Fig. 2a. The position designated as O corresponds to the central point of the device, whereas the positions designated as A and B are situated near the two interfaces connecting the sample and electrodes. The photoresponse characteristics of BiI_3_ under pressure were initially investigated using a xenon lamp as the light source. The light spot size significantly exceeded the active device area, ensuring global illumination of the device. To assess the photoresponse of BiI_3_ at 0.8 GPa, BiI_3_ was subjected to repeated on/off xenon light illumination cycles at various voltages under different incident light powers (Fig. S4a). The photoresponse notably increases with increasing bias from 1 to 15 V, especially at higher incident light powers (*P_in_*). The photoresponse characteristics of BiI_3_ under xenon light illumination from 0.8–31.0 GPa are depicted in Fig. 2b. During compression, the *P_in_* was held constant at 1.1 mW cm^−2^, with the bias set to 5 V. At 0.8 GPa, BiI_3_ exhibits a photocurrent of 96 nA and a responsivity (R) of 0.29 A W^−1^. As the pressure increases, the photocurrent of BiI_3_ gradually decreases, reaching 25 nA at 5.6 GPa. Upon further compression, the photocurrent rapidly increases, reaching ∼225 μA (683 A W^−1^ for R) at 31.0 GPa. This value represents the highest responsivity reported among triiodide systems and exceeds three orders of magnitude higher than the value at 0.8 GPa (Fig. 2c). Furthermore, the enhanced photoelectric properties of BiI_3_ under high pressure are comparable to those observed in RhI_3_ [33] and CsI_3_ [34], and are significantly superior to SbI_3_ [35]. However, the photoresponse speed of BiI_3_ was significantly slower under pressure, which may be attributed to the bolometric effect [36]. Due to variations in crystal structures, the photoresponse characteristics of the two phases of BiI_3_ exhibit inverse regulation under high pressure, and this process is reversible upon decompression. Additionally, the current-voltage (I‒V) characteristic curves of the device were measured to assess the contact of the electrodes with the sample. At 0.8 GPa, the nonlinearity exhibited by the output curve indicates the potential presence of a Schottky barrier between BiI_3_ and the Mo electrode (Fig. S4b). As the pressure increases, the interaction between BiI_3_ and the Mo electrode strengthens, as shown by the linear nature of the I‒V curve, indicating a favorable ohmic state. Importantly, this behavior is maintained even at higher pressures (Fig. S5a, b). The adequate ohmic contact rules out contact resistance effects on the observed photoresponse under pressure [37], confirming that the variation in the photocurrent with pressure is attributable to the intrinsic properties of BiI_3_. The variations in the resistance of BiI_3_ with pressure were derived from the slopes of the I‒V curves. During compression, the resistance of BiI_3_ rapidly decreases as the pressure increases. At 31.0 GPa, the resistance is more than seven orders of magnitude lower than the initial value (Fig. S5c).

**Figure 3.**
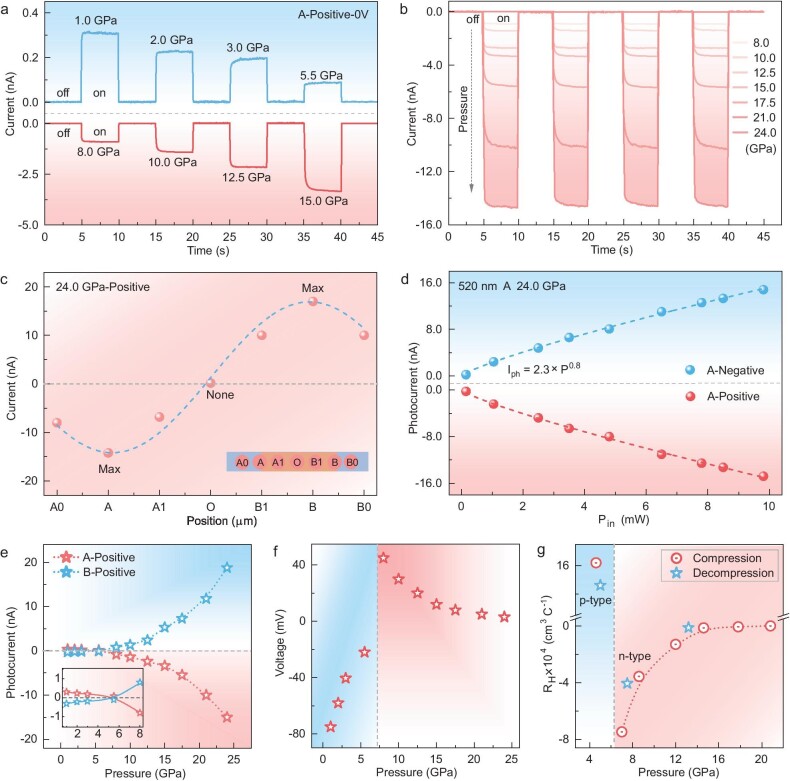
Pressure-induced polarity reversal of self-driven photocurrent in BiI_3_. (a and b) Photoresponse of BiI_3_ under 520 nm laser illumination at position A with zero bias under typical pressures. The light spot diameter of ∼10 μm indicating localized irradiation of the sample. (c) Photocurrent distribution of BiI_3_ at 24.0 GPa as the laser moves between the two electrodes. The distance between two neighboring irradiation points is ∼20 μm. (d) Photocurrent changes of BiI_3_ at 24.0 GPa with varying illumination intensity, extracted from Figure S8. (e) Variation in photocurrent with pressure at illumination positions A and B. (f) Changes in photothermoelectric voltage with pressure at illumination position A. (g) Pressure-dependent Hall coefficient of BiI_3_.

The evolution of society has spurred a growing demand for low-power, high-performance optoelectronic devices, particularly those capable of broad-spectrum responsiveness and self-driven operation [38]. Self-driven photocurrents can be generated via localized irradiation of semiconductors, primarily governed by the photothermoelectric (PTE) effect [30]. To explore the self-driven photocurrent response of BiI_3_ and the effect of pressure on its PTE effect, localized laser illumination was applied to positions A or B of the device, with a spot diameter of ∼10 μm. No bias was applied during the test. Figure S6 displays a photograph of the two-point probe setup used for photocurrent measurements of BiI_3_. In this configuration, the polarity of the photoelectric signal is related to both the illumination position and the conduction type of the material. When the irradiation position remains constant, the carrier type can be determined based on the polarity of the photoelectric signal [39]. Figure S7a, b depict the arrangement of the external testing electrodes showing the positive and negative connections, as explained in the text. The current-time graph during irradiation of position A with a 520 nm laser under positive connection conditions within the pressure range of 1.0–24.0 GPa is depicted in Fig. 3a, b. The device can be adeptly transitioned between the ‘ON’ and ‘OFF’ states by alternately activating and deactivating the laser at intervals. A self-driven photoresponse is detected in the ‘ON’ state at 1.0 GPa, yielding a photocurrent of 0.30 nA. This photocurrent gradually decreases as the pressure increases to 5.5 GPa. Upon further compression, there is a notable inversion in the photocurrent polarity, accompanied by a substantial increase to 15.0 nA at 24.0 GPa, a magnitude 50 times greater than observed at 1.0 GPa. Under negative connection conditions (with irradiation at position A), the current flows in the opposite direction with the same magnitude as under positive connection conditions (Fig. S7c). This suggests that the photogenerated electron-hole pairs are driven by the built-in electric field. A comparable photocurrent trend under high pressures is also observed when the laser irradiates position B (Fig. S7d). As the laser spot moves between the two Mo electrodes, the position-dependent photocurrent of BiI_3_ exhibits an antisymmetric spatial distribution (Fig. 3c). The photocurrent reaches its maximum value when the laser approaches the electrodes, in sharp contrast to the near-zero values observed at the midpoint between the device electrodes. Additionally, the photoelectric signal of the device can be switched between positive and negative as the light moves across its length. The photo-Dember (PD) effect is another plausible explanation for the generation of a photocurrent in the device under zero bias. The photoresponse resulting from the PD effect, which disrupts the electron-hole symmetry, manifests exclusively in the sample-electrode overlapping region [40]. However, since the photoresponse of BiI_3_ is not confined to the contact region, the PD effect can be ruled out as the primary contributing mechanism (Fig. 3c). Therefore, the observed self-driven photocurrent under high pressures, with illumination at positions A and B, can be attributed to the PTE mechanism. The PTE characteristics of BiI_3_ at 24.0 GPa were further investigated under various 520 nm laser illumination intensities (Fig. S8). The BiI_3_ device demonstrates a stable and rapid photoresponse across various light intensities, with a notable increase in photocurrent at higher intensities. This enhancement was attributed to the amplification of the temperature gradient ΔT. The relationship between the photocurrent and illumination intensity is well described by the power function ${{I}_{ph}} = 2.3 \times {{P}^{0.8}}$ (Fig. 3d). Figure 3e summarizes the pressure-dependent photocurrent of BiI_3_ under 520 nm illumination at positions A and B with zero bias, highlighting the variations in both the polarity and magnitude of the photocurrent as pressure changes. ${\mathrm{\Delta }}{{V}_{{\mathrm{PTE}}}}$ signifies the voltage produced through the Seebeck effect in conjunction with ΔT. The polarity of ${\mathrm{\Delta }}{{V}_{{\mathrm{PTE}}}}$ reverses above 5.5 GPa, aligning with the observed trend in photocurrent changes under pressure (Fig. 3f and Fig. S9). This reversal in the polarity of ${\mathrm{\Delta }}{{V}_{{\mathrm{PTE}}}}$ indicates a transition in the conductivity type of BiI_3_. To investigate potential variations in the carrier type and concentration of BiI_3_ under compression, we conducted *in situ* high-pressure Hall measurements using the four-point probe method. Owing to the elevated resistance of BiI_3_ at lower pressures, which exceeds the measurement capacity of our device and thus cannot be evaluated, the initial pressure point documented in Hall measurements is 4.6 GPa. The Hall coefficient (${{{\mathrm{R}}}_{\mathrm{H}}}$) was derived from the field derivative of R_xy_ (Fig. S10). Figure 3g shows the pressure-dependent behavior of ${{{\mathrm{R}}}_{\mathrm{H}}}$. At the initial pressure, ${{{\mathrm{R}}}_{\mathrm{H}}}$ is positive, indicating p-type conduction behavior, consistent with prior research findings [41,42]. Notably, R_H_ transitions from positive to negative around 7.0 GPa, signifying a shift in the dominant charge transport mechanism within BiI_3_, with a transition from hole-type carriers to electron-type carriers. Importantly, this transition is reversible upon decompression. The ability to reversibly switch semiconductor properties within a single compound, transitioning from hole conduction to electron conduction, solely through pressure variations enables the development of novel electronic devices, such as pressure-responsive switching devices. The carrier density (${{{\mathrm{n}}}_{\mathrm{H}}}$), can be determined via the equation ${{{\mathrm{n}}}_{\mathrm{H}}} = \frac{1}{{( {{{{\mathrm{R}}}_{\mathrm{H}}}{\mathrm{e}}} )}}.\ $With increasing pressure, ${{{\mathrm{n}}}_{\mathrm{H}}}$ increases significantly, reaching a value nearly five orders of magnitude higher at 20.8 GPa compared to 4.6 GPa, rising from $3.9 \times {{10}^{13}}$ to $1.6 \times {{10}^{18}}$ cm^−3^ (Fig. S11). The ability to modulate both the polarity and concentration of carriers is crucial in semiconductor fabrication. These results indicate that pressure serves as an effective method for adjusting the carrier properties, showing significant potential for future advancements in 2D electronics engineering.

**Figure 4.**
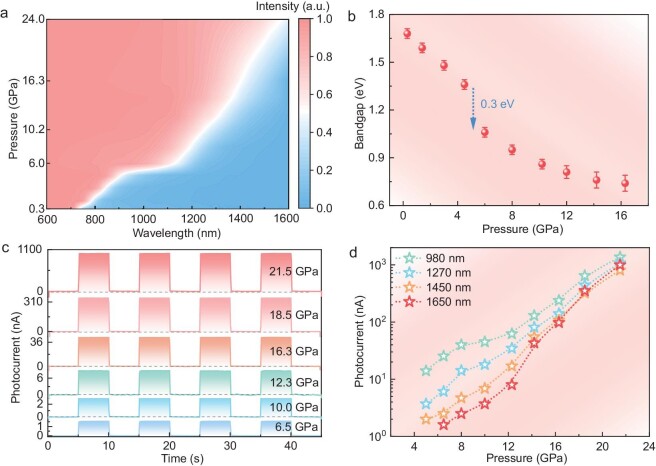
Responsiveness to near-infrared light. (a) 2D projection of the optical absorption spectra of BiI_3_ under high pressures. (b) Pressure-dependent bandgap of BiI_3_. (c) Photocurrent of BiI_3_ at representative pressures under 1650 nm laser illumination at position A with a 5 V bias. (d) Pressure-dependent photocurrent of BiI_3_ under 980 nm, 1270 nm, 1450 nm and 1650 nm laser illumination at position A with a 5 V bias.

The optical absorption spectrum and bandgap play pivotal roles in the practical applications of photoelectric devices. The 2D projection of the optical absorption spectra under high pressures for BiI_3_ is illustrated in Fig. 4a (full-range high-pressure absorption spectra can be found in Fig. S12). Initially, the absorption edge gradually shifts to longer wavelengths as the pressure increases from 0.3 to 4.5 GPa, corresponding to a gradual decrease in the optical bandgap from 1.72 to 1.44 eV. Above 4.5 GPa, a significant redshift occurs, and the bandgap sharply decreases from 1.44 to 1.14 eV, which is consistent with a phase transition. The bandgap continues to decrease with increasing pressure, reaching 0.78 eV at 16.3 GPa (Fig. 4b). With a further increase in pressure, the absorption edge broadens, losing its distinct definition. The functionalities and applications of photodetectors can be substantially enhanced by broadening their spectral response range. Owing to the continuous decrease in its bandgap with increasing pressure, BiI_3_ can exhibit a progressive increase in the intrinsic absorption threshold and generate a photoresponse to longer-wavelength incident light through the photoconductivity mechanism. Furthermore, the PTE mechanism operates independently of the bandgap, providing a significant advantage for further attainment of broadband photoresponses in materials. To explore the influence of pressure on the spectral response range of BiI_3_, *in situ* high-pressure photocurrent measurements were conducted under illumination by lasers of various wavelengths (Fig. 4c, d, and Fig. S13). During the test, the sample was subjected to localized irradiation with a spot size of ∼10 μm, and a bias voltage of 5 V was applied. Below 5.0 GPa, no observable response is detected when either position A or O is irradiated with the 980 nm laser. This result is attributed to the photon energy being less than the bandgap of BiI_3_, which prevents a photoresponse through the photoconductivity mechanism. Additionally, the PTE effect is too subtle for detection. As the pressure increases, photoresponses are sequentially detected under laser irradiation at positions A and O when using wavelengths of 980 nm, 1270 nm, 1450 nm, and 1650 nm. The photoresponse generated when illuminating point A is significantly greater than that observed when illuminating point O (Fig. S14). This improvement is attributed to the combined effects of photoconductivity and the PTE mechanism when light irradiates point A. In contrast, irradiation at point O results solely in the photoconductivity effect. Upon further compression, the photoresponses at all these infrared wavelengths significantly increase, with the photocurrents at 21.5 GPa increasing by two to three orders of magnitude compared to those at the initial pressure. Pressure regulation and the synergistic effects of multiple mechanisms provide valuable insights for designing novel photodetectors with broad-spectrum response capabilities.

**Figure 5.**
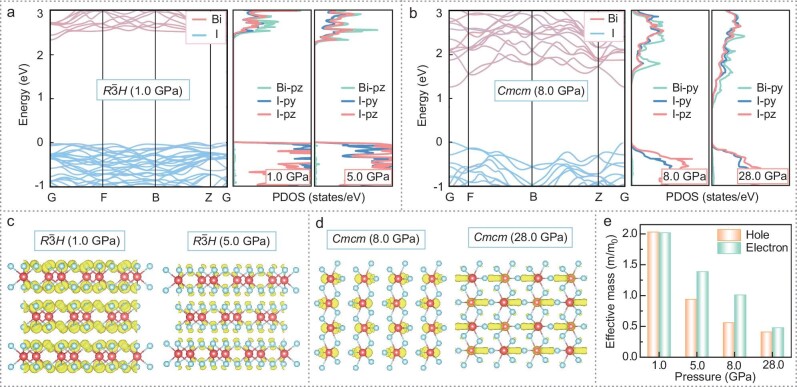
Pressure-induced variations in the electronic properties of BiI_3._ (a) Band structure and PDOS of BiI_3_ in the *R*$\bar{3}$*H* phase at different pressures. (b) Band structure and PDOS of BiI_3_ in the *Cmcm* phase at different pressures. (c) Calculated charge distribution at the VBM of BiI_3_ in the *R*$\bar{3}$*H* phase. (d) Calculated charge distribution at the CBM of BiI_3_ in the *Cmcm* phase. (e) Calculated electron and hole effective masses of BiI_3_ at typical pressures.

To investigate the physical mechanism underlying the dramatic variation in the photoelectric properties upon compression, we conducted first-principles density functional theory-based calculations. The calculated band structure and projected density of states (PDOS) of BiI_3_ manifested as different phases under typical pressures are shown in Fig. 5a, b, and Fig. S15. At 1.0 GPa, the conduction band minimum (CBM) of the *R*$\bar{3}$*H* phase mainly consists of Bi–pz orbitals, whereas the valence band maximum (VBM) is composed primarily of I‒py and I‒pz orbitals. In ns^2^-cation–containing compounds with lone-pair s electrons, the optical transition of electron-hole pair occurs from the occupied anion p orbitals to the unoccupied cation p orbitals, forming a ‘p–p transition’ [43]. This transition is beneficial for achieving optimal band-edge transition intensity. As the pressure increases to 5.0 GPa, the contribution of Bi‒pz orbitals to the CBM gradually decreases, while the I–pz orbitals progressively shift downward toward the CBM (Fig. 5a). This significantly reduces the I‒p to Bi‒p orbital cross-gap transition, thereby decreasing band‒edge light absorption. Furthermore, the carrier charge density distribution (Fig. 5c and Fig. S16) reveals that the partial charge density at the VBM shows an abnormal localization tendency with increasing pressure. This is expected to result in a substantial reduction in hole carrier migration, thereby decreasing photoelectric conversion efficiency. Above 5.5 GPa, BiI_3_ transforms from the *R*$\bar{3}$*H* phase to the *Cmcm* phase, resulting in a significant bandgap reduction. This decreased bandgap enhances the light absorption wavelength range and improves the light capture rate. Compared with the *R*$\bar{3}$*H* phase, the *Cmcm* phase exhibits enhanced dispersion of band-edge states, manifested by the smaller effective mass of carriers and increased carrier delocalization. This effect is further strengthened under compression (Fig. 5b–e and Fig. S16). These changes in electronic structure and photoelectronic properties across the pressure-induced phase transition explain the dramatic increase in the photocurrent upon compression.

The self-driven photoresponse caused by the PTE effect is described by the formula ${{I}_{{\mathrm{ph}}}} = {\mathrm{\Delta }}{{V}_{{\mathrm{PTE}}}}/R$, where ${\mathrm{\Delta }}{{V}_{{\mathrm{PTE}}}}$ denotes the voltage generated via the Seebeck effect and temperature gradient [44]. The magnitude of ${\mathrm{\Delta }}{{V}_{{\mathrm{PTE}}}}$ slightly decreases with increasing pressure in both phases (Fig. 3f). Between 0.8 and 5.6 GPa, the resistance of BiI_3_ gradually decreases, followed by a sharp reduction of nearly five orders of magnitude between 5.6 and 21.5 GPa. Consequently, while a decrease in ${\mathrm{\Delta }}{{V}_{{\mathrm{PTE}}}}$ potentially suppresses the photocurrent of BiI_3_, the significant reduction in the resistance from 5.6–21.5 GPa results in a remarkable increase in the self-driven photocurrent. The polarity reversal of the self-driven photocurrent originates from the switching from p-type to n-type conductivity in BiI_3_. These results indicate that the polarity of the self-generated photocurrent, driven by the PTE effect, can effectively track the conduction type of a material under high pressures. The polarity of this photocurrent, governed by spontaneous carrier movement due to concentration gradients under non-uniform illumination, excludes the influence of the bias voltage. The underlying cause of this shift in conduction type under pressure remains unclear but is generally attributed to intrinsic property alterations related to structural phase transitions. Notably, this transition occurs rapidly, typically within a pressure range of 2–3 GPa. Optical bandgap analysis shows that BiI_3_ retains its semiconducting characteristics, maintaining a bandgap of 1.14 eV even after the conduction type transition. To our knowledge, this study presents the first reported instance of a pressure-induced p–n transition in a semiconductor with such a large bandgap [16,23,24]. The characteristics of BiI_3_, including rapid carrier switching during the semiconductor–semiconductor phase transition and the retention of a suitable bandgap, underscore its significant potential for optoelectronic detection applications.

## CONCLUSION

In summary, we successfully induced dramatic p–n switching during the semiconductor–semiconductor phase transition in BiI_3_ under pressure, accompanied by a significant enhancement in the photoelectric properties. This conductivity transition was confirmed through photocurrent measurements in a zero-bias two-terminal device and Hall coefficient measurements, providing robust evidence of the switching behavior. Additionally, under xenon light illumination, the photocurrent of BiI_3_ at 31.0 GPa increased by more than three orders of magnitude compared with the initial value. Furthermore, the combined effects of the photoconductivity and PTE mechanism enhanced the photoelectric properties and extended the detection bandwidth to 1650 nm under an external bias. This remarkable photoelectric behavior originated from the continuous reduction in the bandgap, increased energy band dispersion, and enhanced charge density at the CBM of BiI_3_ with increasing pressure. Our findings not only contribute to a deeper understanding of semiconductor behavior but also provide valuable insights for the design of logic circuits and the optimization of device performance.

## Supplementary Material

nwae419_Supplemental_File
