# Supplementary material for: Dramatic switchable polarities in conduction type and self-driven photocurrent of BiI3 via pressure engineering
Source: Natl Sci Rev. 2024 Dec 3;12(1):nwae419. doi: 10.1093/nsr/nwae419 (PMC11702651; doi:10.1093/nsr/nwae419)
Supplement: nwae419_Supplemental_File [file nwae419_supplemental_file.zip › Supplementary data.pdf]

Supplementary Information

Dramatic switchable polarities in conduction type and self-driven photocurrent of BiI_3 via pressure engineering

Lei Yue[a,#], Fuyu Tian[b,#], Ran Liu[a], Zonglun Li[a], Ruixin Li[a], Chenyi Li[a], Yanchun Li[c], Dongliang Yang[c], Xiaodong Li[c], Quanjun Li[a,*], Lijun Zhang[b,*], and Bingbing Liu[a,*]

[a]State Key Laboratory of Superhard Materials, Jilin University, Changchun 130012, China;

[b]Key Laboratory of Automobile Materials of MOE and School of Materials Science and Engineering, Jilin University, Changchun 130012, China;

[c]Beijing Synchrotron Radiation Facility, Institute of High Energy Physics, Chinese Academy of Sciences, Beijing 100049, China

***Corresponding authors.** E-mails: liquanjun@jlu.edu.cn; lijunzhang@jlu.edu.cn; liubb@jlu.edu.cn

[#]Equally contributed to this work.

1. Supplementary Methods

X-ray crystallography

In situ ADXRD experiments were conducted at the 4W2 beamline of the Beijing Synchrotron Radiation Facility (BSRF), utilizing a focused monochromatic X-ray beam with a wavelength of 0.6199 Å. Silicone oil was used as the pressure-transmitting medium. Structural refinements were executed via the Rietveld method within the GSAS software.

In Situ photocurrent measurements

The photocurrent response of BiI_3 was evaluated using a two-probe measurement setup fabricated through photolithography within a symmetrical diamond anvil cell (DAC). Two molybdenum (Mo) electrodes were strategically positioned to establish contact with the sample in the chamber without using a pressure-transmitting medium. For the photocurrent measurements of BiI_3 under xenon light illumination, the light spot size greatly exceeds the area of the active device, resulting in global irradiation. The effective irradiation area was the central region between the two electrodes, measuring approximately 300 μm in length and 100 μm in width, for an effective area of 3.0×10^{-4} cm^2. A source meter (Keithley 2461) was used to record the I–T and I–V data. For the measurements of self-driven photocurrent and near-infrared light responsiveness, the photocurrent data were collected using a high-precision photocurrent scanning test microscope system (MStarter 200), and various lasers with different wavelengths (520, 980, 1270, 1450, and 1650 nm) were used as the irradiation source. The light power was recorded using a probe of the power meter placed under the one-side diamond anvil on the original measurement position. During the test, the optical power of the 520, 980, 1270, 1450, and 1650 nm lasers was 9.8 mW, 5.0 mW, 1.7 mW, 1.0 mW, and 0.8 mW, respectively.

Hall measurements

In situ high-pressure Hall measurements were conducted using the standard four-point probe method (with Mo as the electrode material) established via photolithography techniques. These measurements were performed within a screw-pressure-type DAC crafted from a

nonmagnetic Cu-Be alloy operating within a customized multifunctional measurement system (JANIS Research Company Inc.; 0-9 T, Cryomagnetics Inc.). The van der Pauw method was employed to determine all Hall parameters. Two sets of Hall voltages were measured through diagonal 1–3 and 2–4 points, respectively. The final Hall coefficient was obtained by averaging the two results. For data processing, the Hall resistance values from the reverse sequence (from -5 T to 5 T) were subtracted from the original sequence (from 5 T to -5 T) to exclude the effect of asymmetric electrodes, thermal effect, etc.

Absorption spectroscopy

Absorption spectra were acquired over the wavelength range of 400 to 1000 nm using a QE65 Pro spectrometer and over the wavelength range of 910 to 1600 nm using an AvaSpec-NIR512-1.7-HSC-EVO spectrometer. The bandgap was determined using the Tauc plot method by extrapolating the linear portion of the $\alpha^{1/2}$ versus the hv curve, where α is the absorption coefficient, h is the Planck constant, and v is the frequency of the photon.

First-principles calculations

All calculations were performed using plane-wave pseudopotential methods within density functional theory (DFT), implemented in the Vienna Ab initio Simulation Package [1, 2]. Electron–ion interactions were modeled using projector-augmented wave pseudopotentials [3]. The generalized gradient approximation formalism developed by Perdew, Burke, and Ernzerhof [4] was used for the exchange-correlation function. All atomic positions were fully relaxed to minimize total energy, ensuring residual forces on atoms were below 0.05 eV/Å. A kinetic energy cutoff of 500 eV was chosen for the plane-wave basis, and electronic Brillouin zone integration used a k-point grid spacing of $2\pi \times 0.03$ Å^{-1}. To account for long-range van der Waals (vdWs) interactions, the Grimme scheme (DFT+D3) method [5] was applied. The carrier effective masses were calculated with BoltzTrap [6].

References

1. Kresse G, Furthmüller J. Efficiency of ab-initio total energy calculations for metals and semiconductors using a plane-wave basis set. Comput Mater Sci 1996; 6: 15–50.

2. Kresse G, Joubert D. From ultrasoft pseudopotentials to the projector augmented-wave method. Phys Rev B 1999; 59: 1758.

3. Blöchl PE. Projector augmented-wave method. Phys Rev B 1994; 50: 17953.

4. Perdew JP, Burke K, Ernzerhof M. Generalized gradient approximation made simple. Phys Rev Lett 1996; 77: 3865.

5. Grimme S, Ehrlich S, Goerigk L. Effect of the damping function in dispersion corrected density functional theory. J Comput Chem 2011; 32: 1456–65.

6. Madsen GK, Singh DJ. BoltzTraP. A code for calculating band-structure dependent quantities. Comput Phys Commun 2006; 175: 67–71.

2. Supplementary Figures

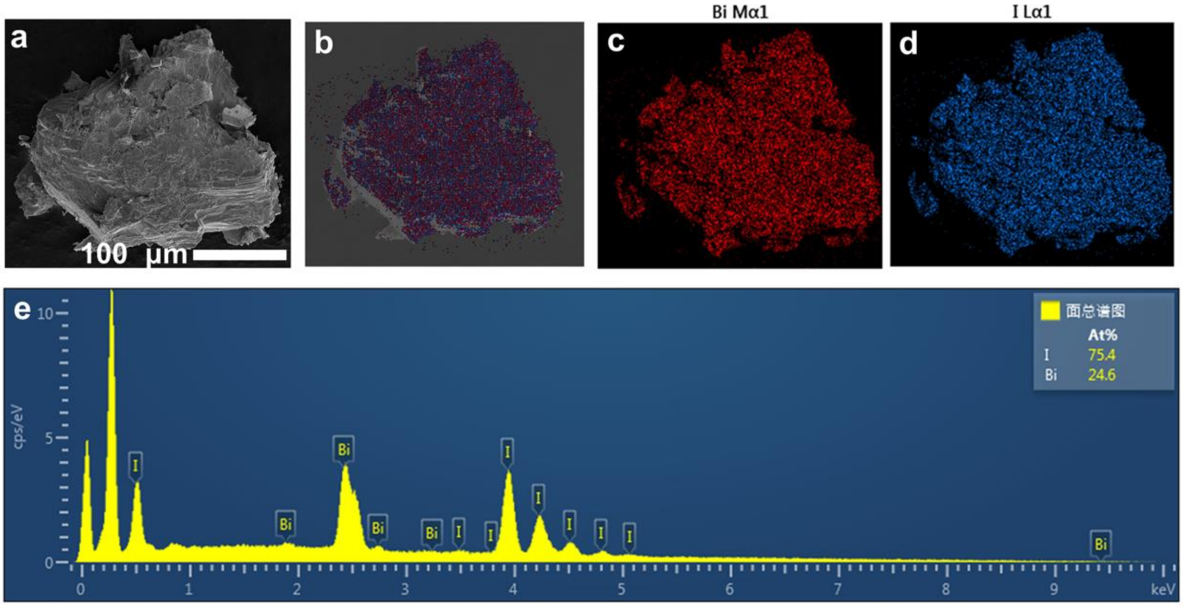

Figure S1. SEM images and EDS results of BiI_3.

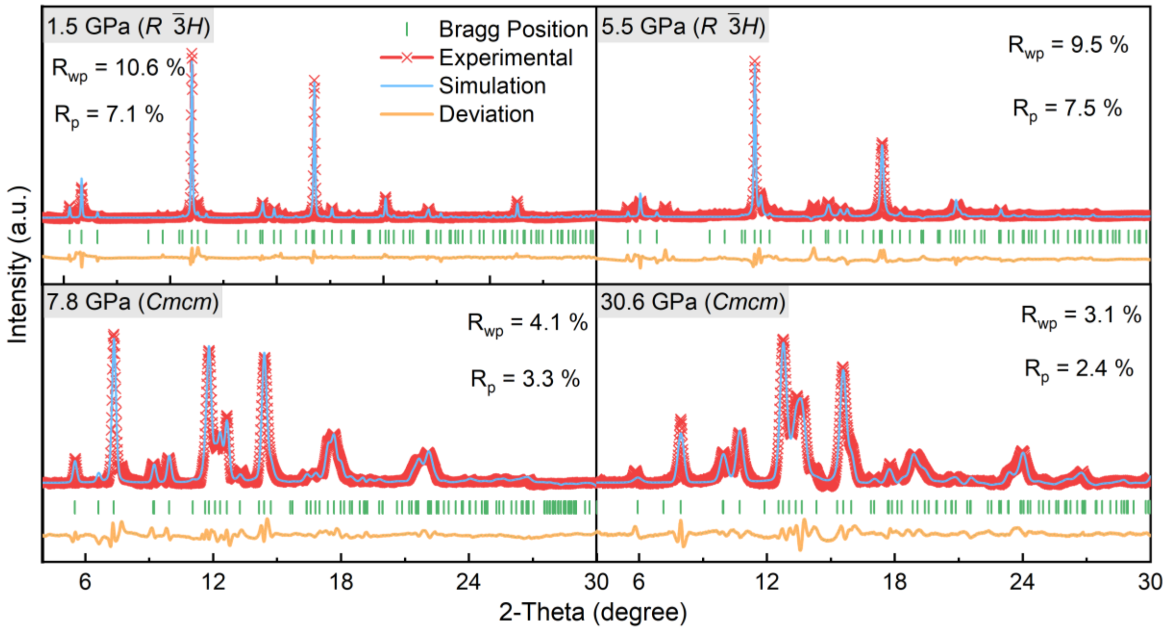

Figure S2. Rietveld refinement results of BiI_3 at representative pressures (1.5 GPa, 5.5 GPa, 7.8 GPa, and 30.6 GPa).

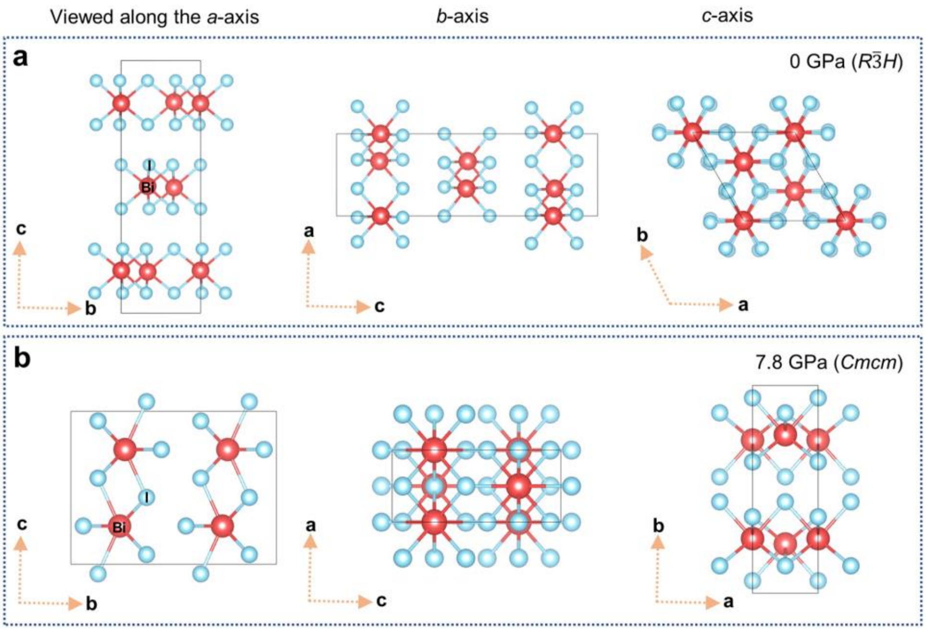

Figure S3. Crystal structures of BiI_3 at ambient pressure and 7.8 GPa, viewed along different crystallographic axes.

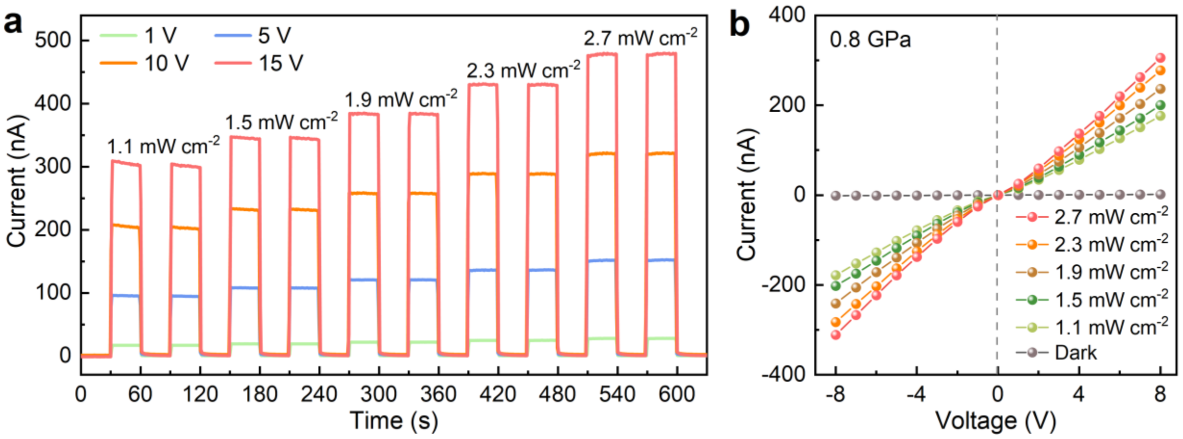

Figure S4. (a) Dependence of the BiI_3 photocurrent on voltage under different incident light power intensities (dark and 1.1–2.7 mW cm^{-2}) at 0.8 GPa. (b) I–V curves of BiI_3 for various incident light powers (dark and 1.1–2.7 mW cm^{-2}) at 0.8 GPa.

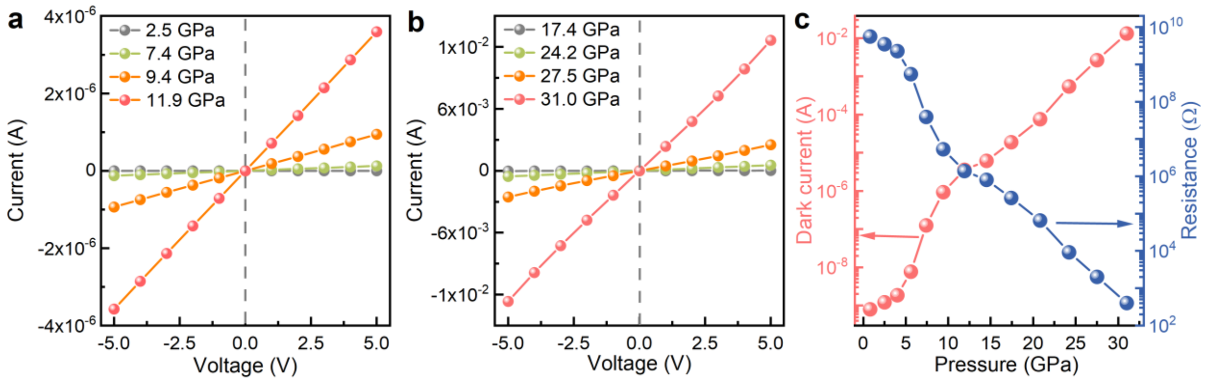

Figure S5. (a, b) I–V curves of BiI_3 under different applied pressures without illumination. (c) The dark current and derived resistance as a function of pressure.

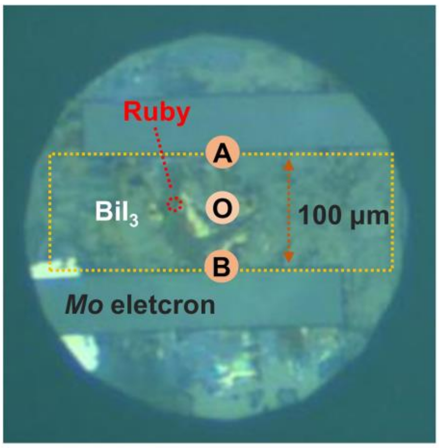

Figure S6. Optical image of the BiI_3 device setup within the DAC.

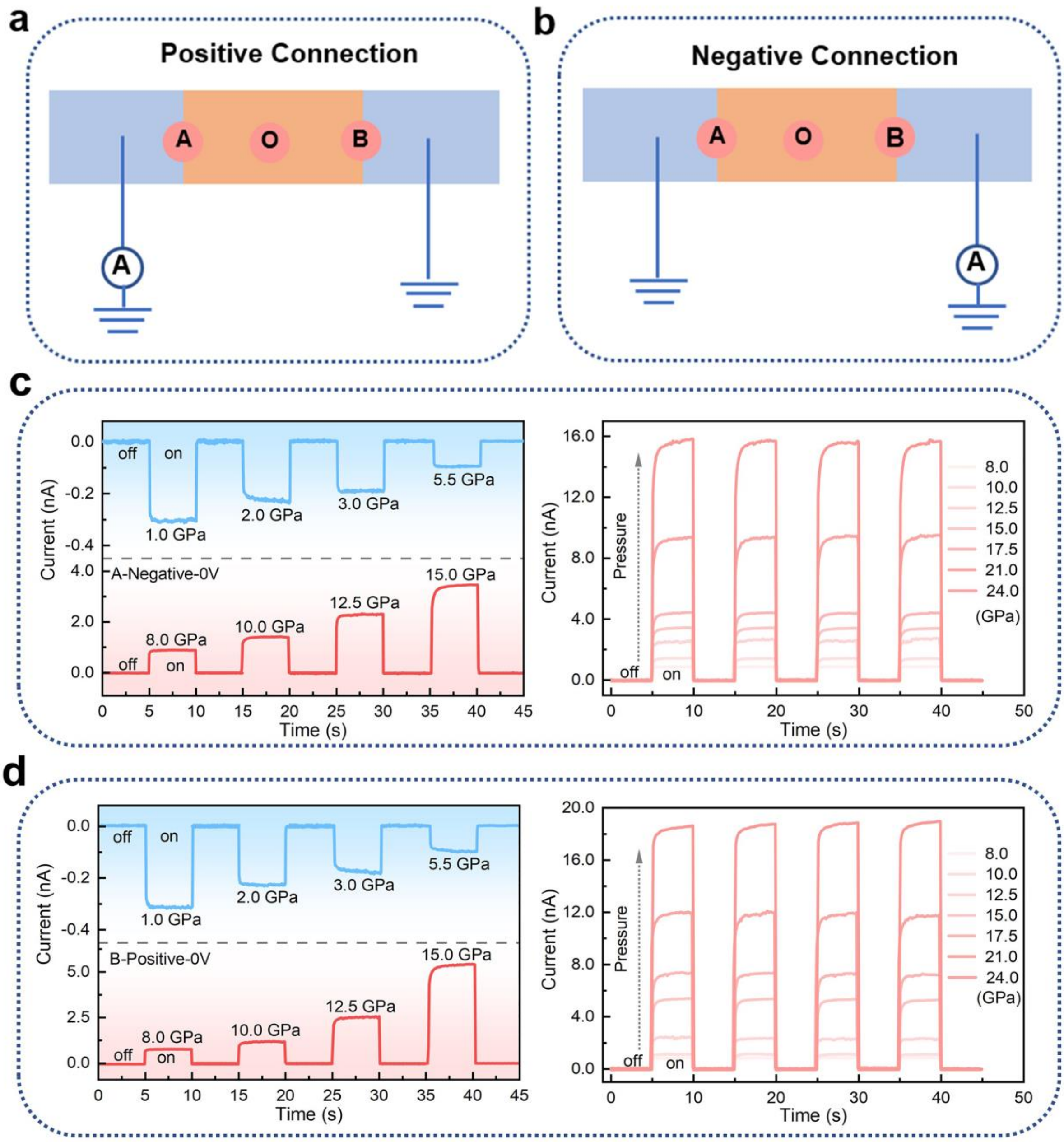

Figure S7. Schematic diagram illustrating the connection of the external electrodes for testing as defined in the text: (a) positive connection and (b) negative connection. (c) Photoresponse characteristics of BiI_3 under 520 nm laser illumination at location A with a negative connection under a 0 V bias. (d) Photoresponse characteristics of BiI_3 under 520 nm laser illumination at location B with a positive connection under a 0 V bias.

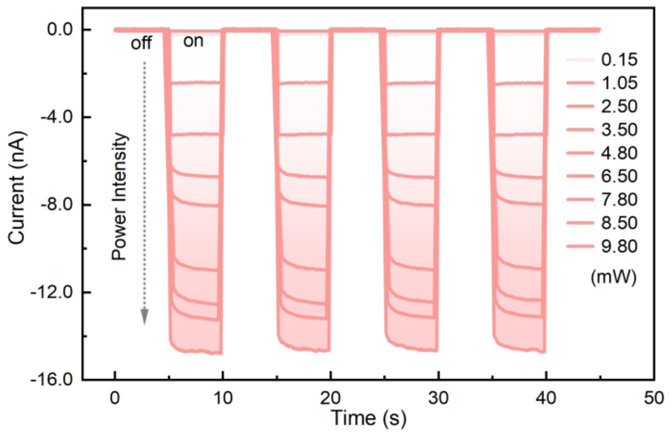

Figure S8. Photoresponse curves of BiI_3 under 520 nm laser illumination with various light intensities at position O at a 0 V bias.

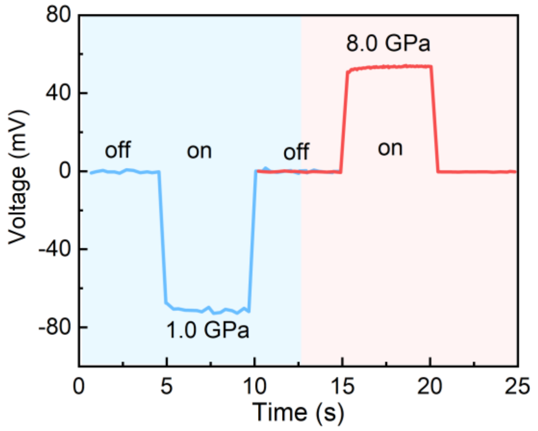

Figure S9. Photothermoelectric voltages of BiI_3 at 1.0 and 8.0 GPa under 520 nm laser illumination at position A.

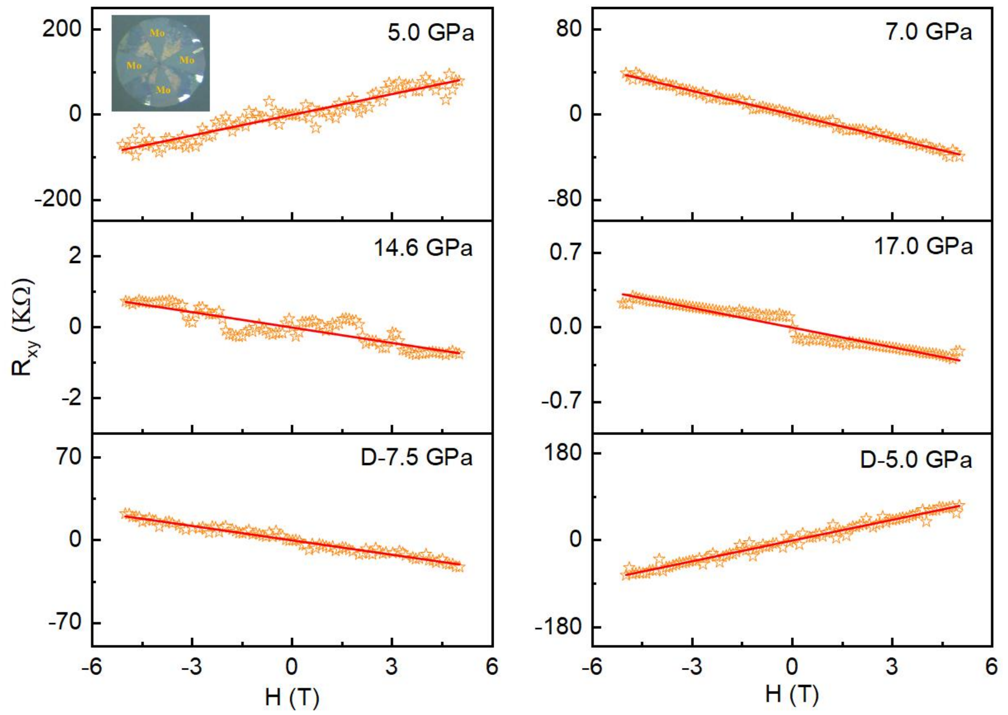

Figure S10. Hall resistance of BiI_3 under typical pressures (4.6 GPa, 7.0 GPa, 14.6 GPa, 17.0 GPa, and released at 7.5 GPa and 5.0 GPa). The data has been processed to eliminate contributions from asymmetric electrodes, thermal effects, and other related factors. The inset shows an optical photograph of the electrode during the measurement

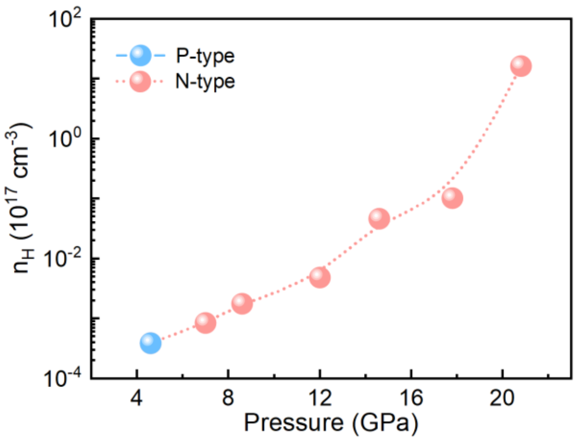

Figure S11. Pressure dependence of the carrier concentration in BiI_3 at 300 K.

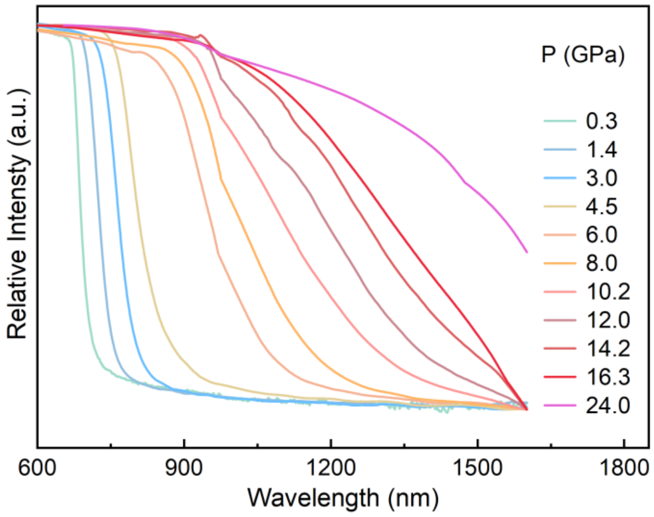

Figure S12. Absorption spectrum of BiI_3 under compression. The data have been smoothed.

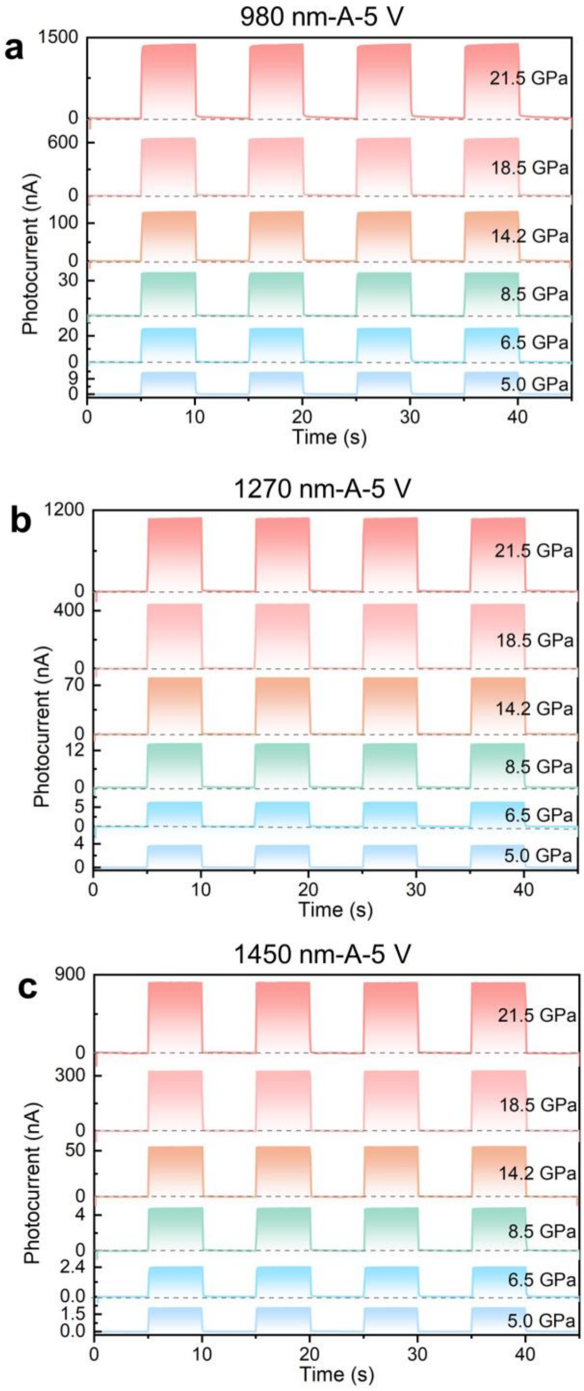

Figure S13. Pressure dependence of the photocurrent of BiI_3 with various laser illuminations at position A under a 5 V bias (a) 980 nm, (b) 1270 nm, and (c) 1450 nm.

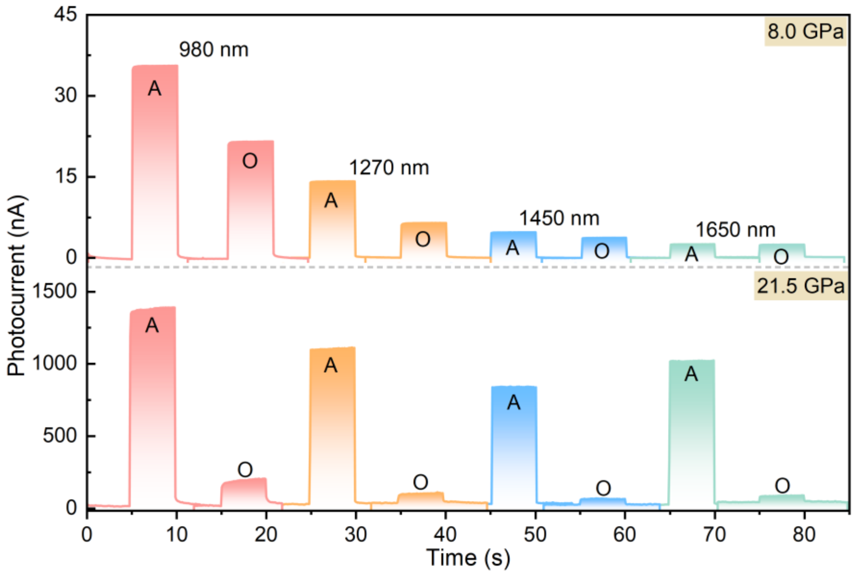

Figure S14. Photocurrent of BiI_3 under near-infrared laser illumination with various wavelengths at positions A and O under a 5 V bias at typical pressures.

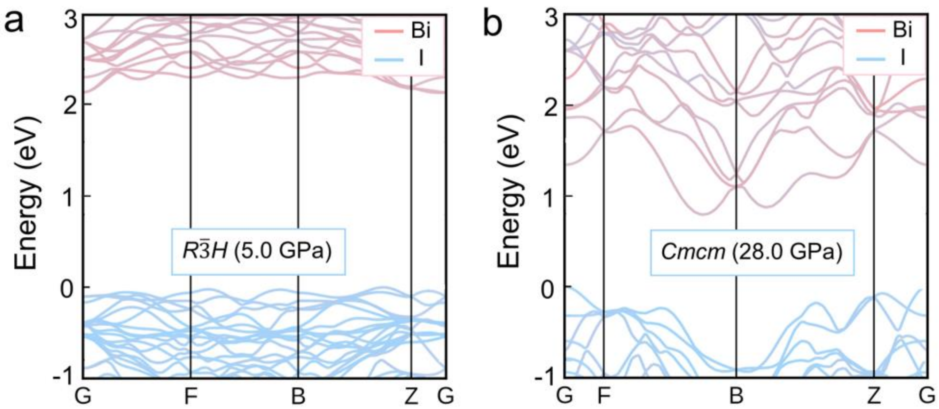

Figure S15. Band structure of BiI_3 at 5.0 and 28.0 GPa.

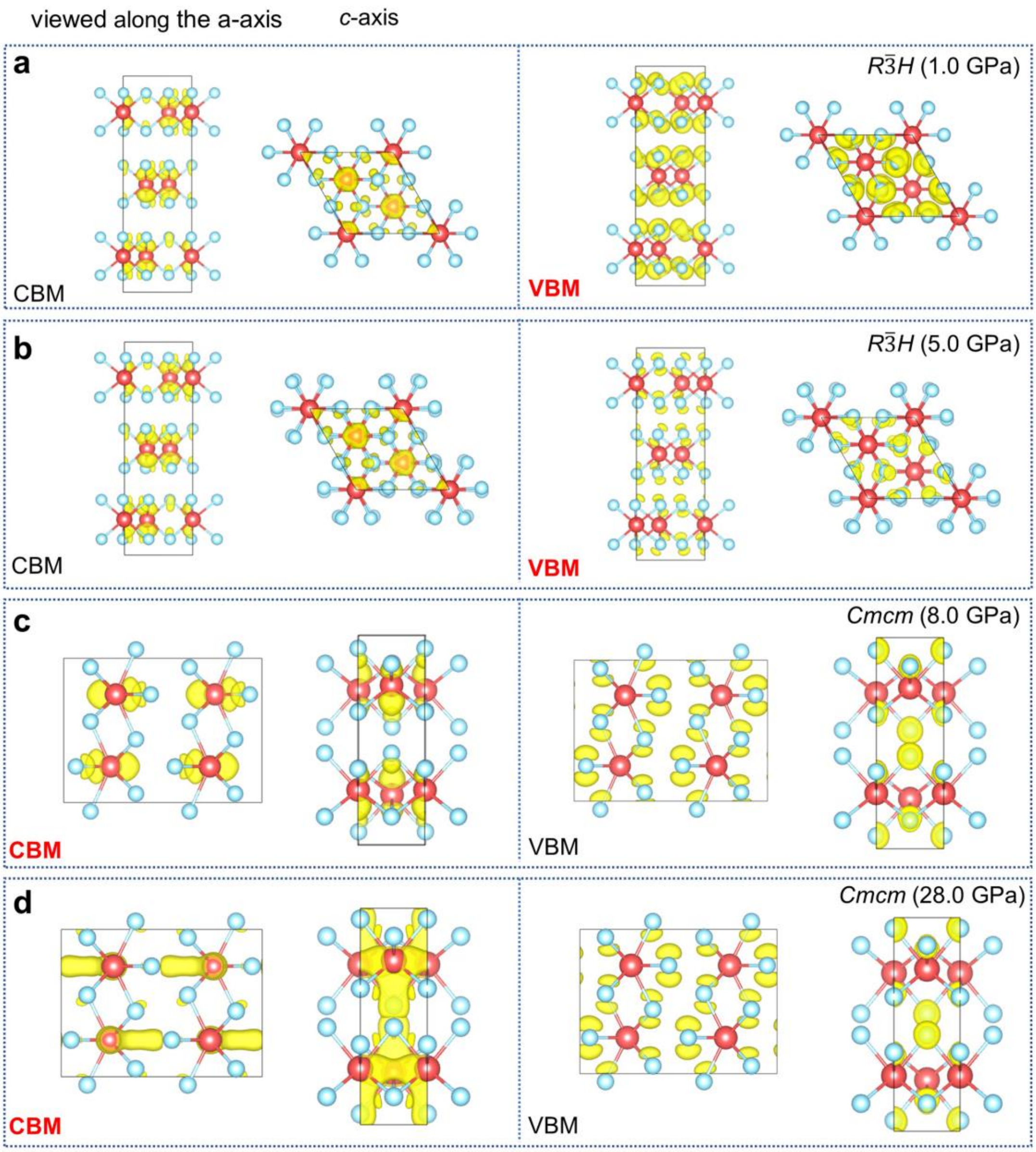

Figure S16. (a, b) Calculated charge distributions at the valence band maximum (VBM) and the conduction band minimum (CBM) of BiI_3 in the $R\bar{3}H$ phase at 1.0 and 5.0 GPa shows a gradual decrease in the partial charge density at the VBM with increasing pressure, whereas the partial charge density at the CBM remains largely unchanged. (c, d) Calculated charge distributions at the VBM and CBM of BiI_3 in the *Cmcm* phase show a gradual increase in the partial charge density at the CBM with increasing pressure, with the partial charge density at the VBM remaining largely unchanged.

Table S1. Crystallographic data of BiI_3 at selected pressures.

Pressure (GPa)	*a* (Å)	*b* (Å)	*c* (Å)	*V* (Å)
0 ($R\bar{3}H$)	7.5250 (70)	7.5250 (70)	20.7310(60)	1016.63(8)
1.5 ($R\bar{3}H$)	7.3657(2)	7.3657(2)	20.1730(20)	947.85(8)
2.6 ($R\bar{3}H$)	7.2506(3)	7.2506(3)	19.9790(40)	909.61(15)
4.0 ($R\bar{3}H$)	7.1536(3)	7.1537(3)	19.7130(40)	873.66(16)
5.5 ($R\bar{3}H$)	7.0941(5)	7.0941(5)	19.4251(50)	846.59(18)
7.8 (*Cmcm*)	4.0374(5)	12.8948(21)	9.6882(9)	504.39(11)
9.5 (*Cmcm*)	4.0023(5)	12.7451(27)	9.5724(9)	488.29(12)
12.4 (*Cmcm*)	3.9606(7)	12.5600(10)	9.4508(10)	470.14(14)
14.8 (*Cmcm*)	3.9566(4)	12.3560(10)	9.3085(6)	455.07(14)
17.2 (*Cmcm*)	3.9509(5)	12.2829(21)	9.1848(9)	445.73(9)
19.8 (*Cmcm*)	3.9310(6)	12.2220(40)	9.0825(13)	436.35(17)
25.6 (*Cmcm*)	3.8237(14)	12.0020(80)	9.0033(23)	411.57(25)
30.6 (*Cmcm*)	3.7421(5)	11.9680(50)	8.9319(12)	400.00(16)
