# Supplementary material for: Dramatic switchable polarities in conduction type and self-driven photocurrent of BiI3 via pressure engineering
Source: Natl Sci Rev. 2024 Dec 3;12(1):nwae419. doi: 10.1093/nsr/nwae419 (PMC11702651; doi:10.1093/nsr/nwae419)
Supplement: nwae419_Supplemental_File [file nwae419_supplemental_file.zip › Teaser text.docx]

This study reports a dramatic reversible p−n switching in BiI3 under pressure and proposes a feasible method to determine the conduction type of materials via photoelectric measurements.
